# Effects of Home-Based Robotic Therapy Involving the Single-Joint Hybrid Assistive Limb Robotic Suit in the Chronic Phase of Stroke: A Pilot Study

**DOI:** 10.1155/2019/5462694

**Published:** 2019-03-18

**Authors:** Koichi Hyakutake, Takashi Morishita, Kazuya Saita, Hiroyuki Fukuda, Etsuji Shiota, Yasuki Higaki, Tooru Inoue, Yoshinari Uehara

**Affiliations:** ^1^Department of Rehabilitation Medicine, Fukuoka University Hospital, Fukuoka, Japan; ^2^Department of Neurosurgery, Faculty of Medicine, Fukuoka University, Fukuoka, Japan; ^3^Graduate School of Sports and Health Sciences, Fukuoka University, Fukuoka, Japan; ^4^Center for Preventive, Anti-Aging, and Regenerative Medicine, Fukuoka University Hospital, Fukuoka, Japan

## Abstract

**Introduction:**

Robotic therapy has drawn attention in the rehabilitation field including home-based rehabilitation. A previous study has reported that home-based therapy could be more effective for increasing upper limb activity than facility-based therapy. The single-joint hybrid assistive limb (HAL-SJ) is an exoskeleton robot developed according to the interactive biofeedback theory, and several studies have shown its effectiveness for upper limb function in stroke patients. A study of home-based robotic therapy has shown to enhance rehabilitation effectiveness for stroke patient with a paretic upper limb. However, home-based therapy involving a HAL-SJ in stroke patients with paretic upper limbs has not been investigated. The present study aimed to investigate paretic upper limb activity and function with home-based robotic therapy involving a HAL-SJ in stroke patients.

**Materials and Methods:**

A home-based robotic therapy program involving a HAL-SJ was performed for 30 min per session followed by standard therapy for 30 min per session, 2 times a week, for 4 weeks (i.e., completion of all 8 sessions involved 8 h of rehabilitation), at home. After the intervention, patients were followed up by telephone and home visits for 8 weeks. The paretic upper limb activity and function were assessed using the Motor Activity Log (MAL; amount of use (AOU)), arm triaxial accelerometry (laterality index (LI)), the Fugl–Meyer assessment (FMA), and the action research arm test (ARAT), at baseline and week 4 and week 12 after the start of training.

**Results:**

The study included 10 stroke patients (5 men; mean age, 61.1 ± 7.1 years). The AOU scores and LI significantly improved at week 4 after the start of training (*p*<0.05). However, no significant changes were observed in the LI at week 12 (*p*=0.161) and the FMA scores at both week 4 and week 12 (*p*=0.059 and* p*=0.083, respectively). The ARAT scores significantly improved at both week 4 and week 12 (*p*<0.05).

**Conclusion:**

Home-based robotic therapy combined with conventional therapy could be a valuable approach for increasing paretic upper limb activity and maintaining paretic upper limb function in the chronic phase of stroke.

## 1. Introduction

Many stroke patients (50–70%) experience long-term upper limb dysfunction [1] and decreased use of the paretic upper limb in daily life [2]. According to the findings of neuroplasticity in an animal study, high-intensity rehabilitation is considered important to promote physiological remapping of the area adjoining to the primary motor cortex [3]. Consistent with the theory of learned nonuse, recovery of motor function after stroke is use-dependent [4]. The principles of stroke rehabilitation include goal setting, high-intensity practice, multidisciplinary team care, and task-specific training [5]. There are several approaches of rehabilitation therapy after stroke, such as magnetic therapy, botulinum toxin (BTX-A) therapy, and robotic therapy [6–8]. In addition, a rehabilitation program often provides facility-based and home-based therapies.

With regard to the environment of rehabilitation, an animal study showed that an enriched environment combined with task-specific rehabilitative therapy can promote brain plasticity and enhance functional outcomes [9]. However, there might be a difference between the activities performed in the rehabilitation unit and the activities actually performed at home. A previous study reported that each activity of daily living (ADL) was less well performed at home than in the rehabilitation unit in 25–45% of cases [10]. Thus, transfer of the rehabilitation technique from the hospital to the home is important [11]. A previous study reported that the home-based therapy has the advantage of a familiar home environment with real world activities [12].

With regard to method of rehabilitation, robotic therapy is expected to reduce medical cost and human resource use. Although a previous systematic review reported that robotic therapy improves paretic upper limb function [13], robotic therapy with an end-effector robot was shown to result in a limited increase in the ADL score and limited use of the paretic upper limb in stroke patients [14]. On the other hand, therapy involving an exoskeleton portable robot (hybrid assistive limb (HAL; Cyberdyne Inc., Ibaraki, Japan), including a single-joint hybrid assistive limb (HAL-SJ)) was shown to be effective for improving motor function and use of the paretic upper limb in the chronic phase of stroke [15]. HAL is a wearable-type robot developed based on the “interactive biofeedback” theory [16]. The power-assist of the HAL is triggered by the bioelectrical signal (BES) sensed from a muscle. The HAL assists the voluntary movements of the paretic limbs and provides the successful movements to the brain on the sensory feedback [17]. With the biofeedback effect of the HAL, the task-related cortical activity can be directly increased [18], and the HAL has shown efficacy among stroke patients in several studies [15, 16, 18, 19].

Thus, both home-based therapy and robotic therapy have, respectively, shown their efficacy. If they are combined, there is a possibility to improve the performance of upper limb function of stroke patients by taking advantage of the strengths of each of them. In fact, a previous study reported that home-based therapy could increase upper limb activity and improve upper limb function. In addition, there have been several reports on home-based therapy involving robots in patients with paretic upper limbs after stroke [20–24]. Among these, the portable robot suit for upper limb, HAL-SJ can be used in combination with conventional therapy and may be adapted for home-based therapy. Thus far, home-based therapy involving the use of a HAL-SJ in stroke patients with paretic upper limbs has not been investigated.

We, therefore, hypothesized that home-based therapy and robotic therapy with a HAL-SJ would enhance the effect of rehabilitation for increasing upper limb activity and upper limb function in patients with chronic stroke. Thus, the present study aimed to investigate paretic upper limb activity and upper limb function with home-based therapy involving a HAL-SJ after stroke.

## 2. Materials and Methods

**Figure 1:**
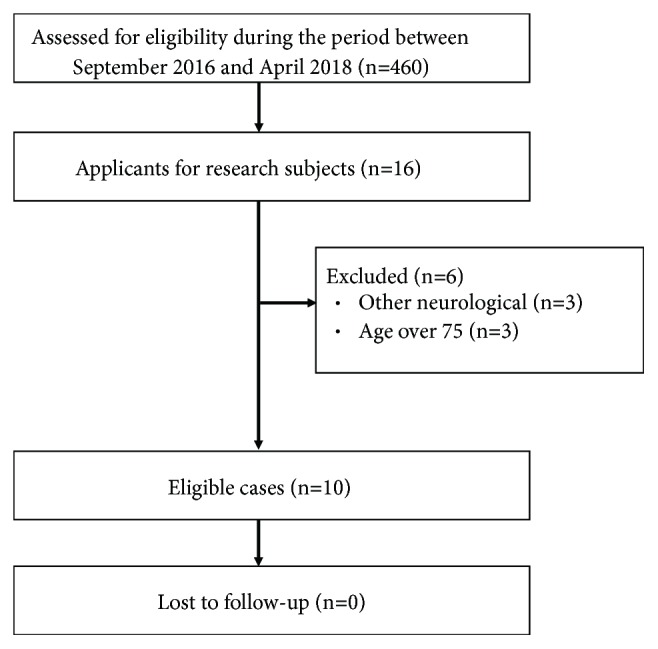
Study flowchart.

We targeted 460 patients with chronic stroke (stroke onset >6 months) who visited our hospital for outpatient consultation in this study during the period between September 2016 and April 2018. Sixteen participants wished to participate in the research by looking at the pamphlet recruiting research participants. The exclusion criteria were as follows: age <40 years or >75 years, inability to follow directions owing to cognitive impairment, and presence of complete paralysis, severe pain, moderate joint disorder, or joint contracture in the upper extremities. We excluded patients with age over 75 (n=3) or other neurological disorder (n=3). Finally, we included 10 patients (5 men and 5 women; mean age, 61.1 ± 7.1 years; mean interval from stroke onset, 25.2 ± 20.1 months; all right handed) (Figure 1).  Table 1 summarizes the demographic and neurological characteristics of the paretic patients. The present study was conducted according to the Declaration of Helsinki. Written informed consent for examination was obtained from all patients, and the Ethical Committee of Fukuoka University approved the present study (IRB No. 16-7-08; UMIN ID: 000026678).

**Figure 2:**
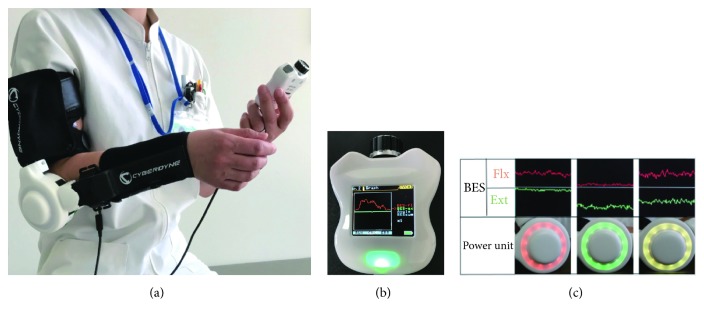
*Single-joint hybrid assistive limb (HAL-SJ). (a) HAL-SJ attachment. (b) Controller showing the bioelectrical signal (BES). Monitor indicating the flexor and extensor muscles. (c) The power unit has visual feedback function in the elbow joint on the lateral side.* The light-emitting diode shows red, green, or yellow depending on upper limb coordination.

**Figure 3:**
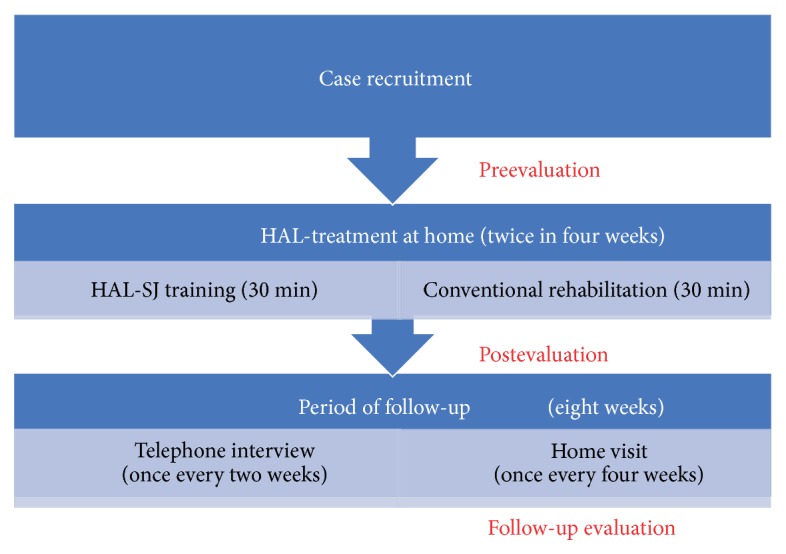
Study design of home-based therapy with robot-assisted rehabilitation.

The study patients underwent HAL-SJ training and conventional therapy. The HAL-SJ system used in this study included an exoskeleton robot with a small power unit and 2 attachments for the forearm and upper arm. The robot is compact and portable (weight, 1.3 kg). Its movement can be triggered by the biceps and triceps. The controller has a monitor that displays the electrical signals from the trigger muscles. A therapist can adjust the assist gain and the flexion/extension balance for each patient using the controller. The movements of the upper limb are displayed waveform of the flexor and extensor muscles on the controller monitor. The controller is held in the hand of the therapist, and the monitor can be shown to the therapist or patient. Other visual feedback function as via a light-emitting diode (LED) placed at the joint of the robot suit is also provided motion statuses to the therapist and patient during the training (Figure 2) [18]. In HAL-SJ training, each patient repeated extension and flexion movements of the elbow joint. As necessary, the therapist and patient confirmed motion statuses with the controller monitor or LED and selected the one which is easy to see, either the controller monitor or LED. After the movements of the HAL-SJ became a rhythmic coordination pattern, the movements of the upper limb were changed in the reaching direction and speed according to the individual rehabilitation goal or motor learning. Conventional therapy involved task-specific ADL training (to provide training on the use of the paretic upper limbs at home). HAL-SJ training was performed for 30 min per session followed by conventional therapy training for 30 min per session, 2 times a week, for 4 weeks (i.e., completion of all 8 sessions involved 8 h of rehabilitation), at home. After the intervention, patients were followed up by telephone and home visits for 8 weeks (Figure 3).

Clinical evaluations were performed at baseline and week 4 and week 12 after the start of training (Figure 3). Before starting the training, patients shared their intended goals, such as lifting a glass to the mouth smoothly and handling a clothes zipper, with an occupational therapist. Baseline use of the paretic upper limbs in ADLs was evaluated with the Motor Activity Log (MAL; mean range 0–5; amount of use (AOU)) [25] and accelerometry (laterality index (LI)). MAL-AOU is evaluated according to the “evaluation manual” by asking 14 questions about the use of the paretic limbs in ADLs and assigning scores of 0–5 for the activities according to the AOU [25]. To evaluate paretic upper limb activity, patients wore 2 activity monitors on each wrist for 24 h, except when bathing [26–28]. Generally, the LI is computed using the following formula: (paretic upper limb numeric accelerometry value – other side upper limb numeric accelerometry value) / (paretic upper limb numeric accelerometry value + other side upper limb numeric accelerometry value). The LI has scores from -1 to +1, and a high LI indicates a high ratio of using the paretic upper limb in ADLs [29, 30].

The present study used the Device Arm Triaxial Accelerometry system (UW-301BT Life Log, Hitachi, Tokyo), which includes a 3-axis accelerometer and an analog-to-digital converter with a 20 Hz sampling rate [31]. The device is relatively small (width, 20 mm; length, 39 mm; height, 14 mm; weight, 20 g). Acceleration data for each axis were saved for computer analysis. After removing the signal caused by the constant offset associated with gravity and external vibration using a band-pass filter (1.0–5.0 Hz), large acceleration was obtained for each axis. The resultant signal was integrated to calculate data for each minute, which is referred to as the movement count [26, 31]. We also assessed upper limb function using the Fugl–Meyer assessment- (FMA-) upper extremity (FMA-UE; score range, 0–66) [32] and action research arm test (ARAT; score range, 0–57) [33].

The Wilcoxon signed-rank test was used to compare the AOU, LI, FMA-UE, and ARAT scores between pre- and postintervention and between preintervention and follow-up. All analyses were performed using SPSS version 21.0 (IBM Corp., Armonk, NY, USA). The statistical threshold was set to* p* < 0.05. The effect size index* d* was also calculated.

## 3. Results

The study included 10 patients. All patients were able to use the devices and complete the training and follow-up. The demographic and clinical characteristics are shown in Table 1. Of the 10 patients, 5 had hemorrhagic stroke and 5 had ischemic stroke. Additionally, 5 had right upper limb paralysis and 5 had left upper limb paralysis.

**Figure 4:**
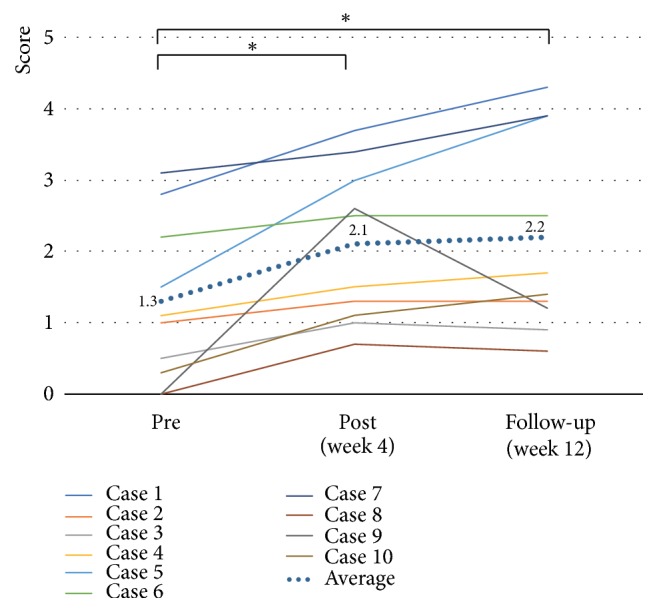
*Effects of home-based robotic therapy with a single-joint hybrid assistive limb according to the amount of use.* The line graphs demonstrate the mean amount of use score (0–5). Pre- versus postintervention and preintervention versus follow-up. ^*∗*^*p* < 0.05 (Wilcoxon signed-rank test).

**Figure 5:**
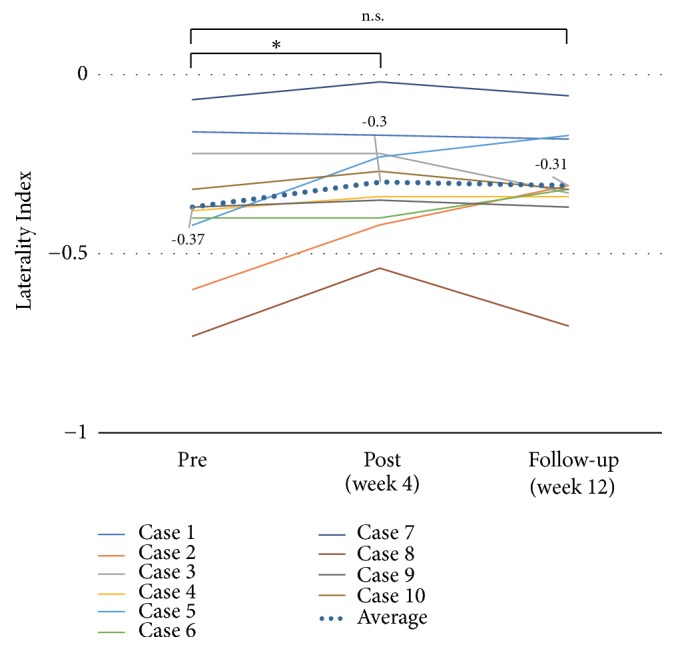
*Effects of home-based robotic therapy with a single-joint hybrid assistive limb according to the laterality index.* The line graphs demonstrate the mean laterality index (-1 to +1). Pre- versus postintervention and preintervention versus follow-up. ^*∗*^*p* < 0.05 (Wilcoxon signed-rank test).

**Figure 6:**
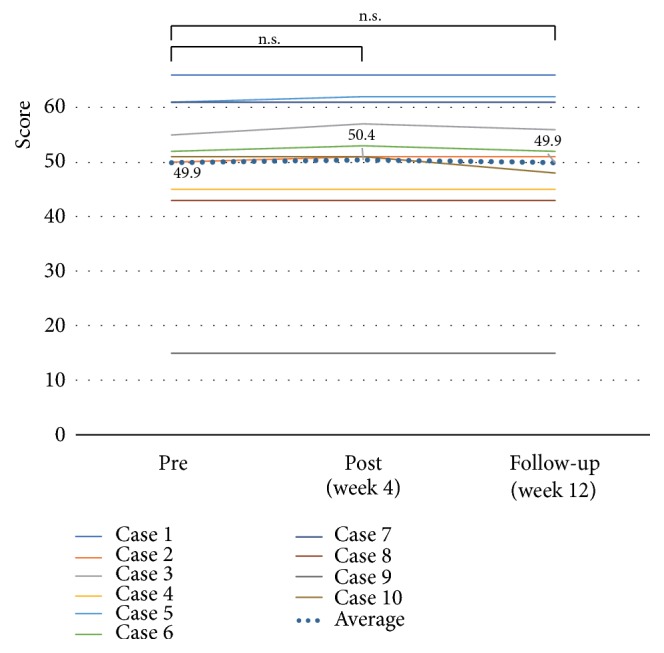
*Effects of home-based robotic therapy with a single-joint hybrid assistive limb according to the Fugl–Meyer assessment.* The line graphs demonstrate the mean Fugl–Meyer assessment-upper extremity score (range, 0–66). Pre- versus postintervention and preintervention versus follow-up. ^*∗*^*p* < 0.05 (Wilcoxon signed-rank test).

**Figure 7:**
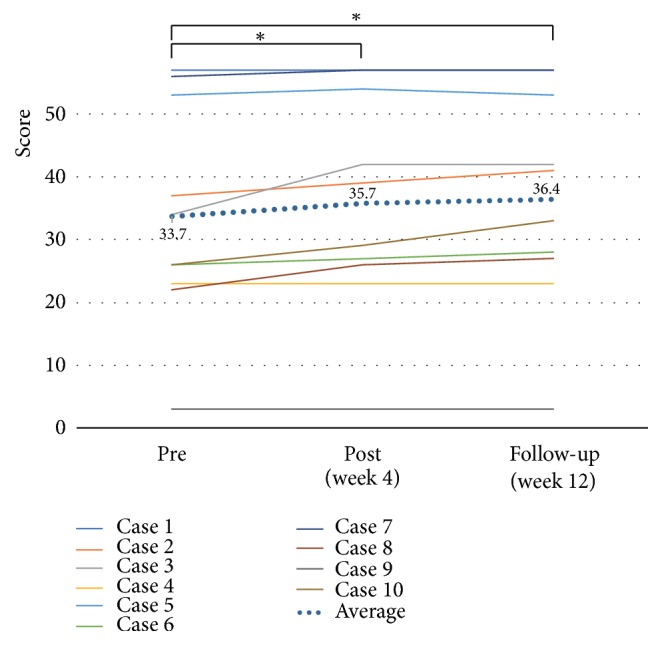
*Effects of home-based robotic therapy with a single-joint hybrid assistive limb according to the action research arm test.* The line graphs demonstrate the mean action research arm test score (range, 0–57). Pre- versus postintervention and preintervention versus follow-up. ^*∗*^*p* < 0.05 (Wilcoxon signed-rank test).

The clinical outcomes are shown in Table 2. With regard to paretic upper limb use and paretic upper limb activity, the MAL-AOU score significantly improved at both week 4 and week 12 after the start of training (2.1 ± 1.0,* p* = 0.005,* d* = 0.76, and 2.2 ± 1.3,* p* = 0.005,* d *= 0.75, respectively) when compared to the baseline value (1.3 ± 1.1) (Figure 4). The LI significantly improved at week 4 (-0.30 ± 0.1,* p* = 0.017,* d* = 0.44) when compared to the baseline value (-0.37 ± 0.2); however, there was no significant change at week 12 (-0.31 ± 0.2,* p* = 0.161,* d* = 0.30) (Figure 5). With regard to motor function, the mean total FMA-UE score did not significantly change at both week 4 and week 12 (50.4 ± 13.7,* p* = 0.059,* d* = 0.04, and 49.9 ± 13.7,* p* = 0.083,* d* = 0.00, respectively) when compared to the baseline value (49.9 ± 13.5) (Figure 6). On the other hand, the mean ARAT score showed significant improvements at both week 4 and week 12 (35.7 ± 16.5,* p* = 0.017,* d* = 0.12, and 36.4 ± 16.3,* p* = 0.028,* d* = 0.17, respectively) when compared to the baseline value (33.7 ± 16.5) (Figure 7).

## 4. Discussion

The present study found that home-based robotic therapy with a HAL-SJ was associated with increased paretic upper limb activity and maintained upper limb function. To our knowledge, this is the first pilot study to find out the potential of this novel home-based program involving a robot-assisted approach.

With regard to paretic upper limb activity, effect sizes of the MAL-AOU was medium to large and accelerometer was small to medium. The MAL-AOU score improved at both week 4 and week 12. The minimal clinically important difference (MCID) for this score has been reported to be 0.5 following chronic stroke [34]. In the present study, the changes at both time points were over 0.5; thus, these changes were considered clinically meaningful during the period of the program. On the other hand, upper limb activity assessed with an accelerometer has been reported to be significantly correlated with the frequency of paretic limb use and upper limb function [26]. In addition, several researches showed that the MAL is significantly correlated with wrist-worn accelerometer counts in the home before and after constraint-induced movement therapy [35]. However, LI at week 12 did not significantly change. The discrepancy may have resulted because the LI was measured after only 24 hours of accelerometry recording, whereas the MAL-AOU assesses the spontaneous activity over a longer period. In addition, it is possible that the accelerometers inhibited spontaneous paretic arm use. A previous study showed that home-based stroke rehabilitation demonstrated better improvements on the MAL-AOU than in the clinic-based group [20]. The home-based therapy has the advantage of a familiar home environment with real world activities without transfer of the rehabilitation technique from the hospital to the home [12]. Therefore, our home-based program may enhance increasing upper limb activity in chronic stroke patients with paretic upper limbs.

According to the findings of neuroplasticity in an animal study, high-intensity rehabilitation is considered important to promote neuroplastic changes in areas adjoining to the primary motor cortex [3]. Additionally, considering the theory of learned nonuse, recovery of motor function after stroke is use-dependent [4]. Thus, the significant increase in paretic upper limb activity indicated that recovery is use-dependent, and, therefore, an increase in upper limb activity will help prevent and improve dysfunction.

With regard to upper limb function outcomes, the ARAT score significantly improved at both week 4 and week 12 after the start of training. A previous study reported that home-based therapy using robotic assist and biofeedback would increase upper limb function in chronic stroke [21]. In this respect, our results of upper limb function may have supported our hypotheses. The ARAT can be used to assess paretic upper limb performance, such as ADLs [33]. The patients mostly had mild upper limb paralysis, and the rehabilitation goals of ADL tasks were considered to be similar to the characteristics of the ARAT, such as lifting a glass to the mouth smoothly and handling a clothes zipper. Additionally, with regard to brain function, combination therapy involving a HAL-SJ could effectively alter motor-related cortical activity, as demonstrated by clinical functional imaging in previous studies [15, 18]. Similarly, the effect of brain function activated by HAL-SJ training possibly shifted to that with conventional therapy during home visits for 4 weeks. However, compared with the reported MCID of the ARAT score (5.7 points) following chronic stroke [34], our ARAT score showed a small change. The FMA score indicated maintenance of paretic upper limb function, and this result was associated with the ceiling effect of the FMA for measuring the degree of paralysis. We suggested that the amount of training and frequency of exercise need to be greater to improve paretic limb function during the program.

The HAL-SJ is a lightweight system and can be used easily anywhere. In addition, the HAL-SJ supports the voluntary movements of the paretic upper limb with biofeedback [15, 18]. In this respect, we consider that a HAL-SJ might effectively assist upper limb training and lead to standardized training. However, we believe that it is difficult to show effective improvement of upper limb function only in the intervention period with current medical systems and human resources. The extent of robotic therapy should be increased and upper limb activity limitations should be efficiently addressed by therapists. If the HAL-SJ system is improved with a self-attachable robot in the future, patients who have experienced stroke will be able to maintain upper limb function and perform long-term training.

The present study had some limitations. First, there was no control group. Prior to the study, we initially tried to set up a control group but abandoned this when faced with ethical problems. Second, this study needed to assess the accelerometry over a longer period.

Thus, further studies, such as randomized controlled trials, are required to accumulate cases to demonstrate the effectiveness of home-based robot-assisted therapy.

Training that combines robot-assisted therapy with visiting rehabilitation with a focus on paretic upper limb activity and upper limb function may help increase paretic upper limb activity and maintain upper limb function. We believe that our findings will help in the development of novel methods that utilize robots in home-based therapy.

## 5. Conclusion

The present study found that home-based robot-assisted therapy along with conventional therapy is a valuable approach to increase paretic upper limb activity and maintain paretic upper limb function in the chronic phase of stroke.

## Figures and Tables

**Table 1:** Demographic and clinical characteristics.

Case	Age	Sex	Handedness	Stroke diagnosis	Lesion location	Interval from onset
	(years)					(months)
1	48	F	R	Ischemic	Left ACA	8
2	68	F	R	Hemorrhagic	Right subcortical region	28
3	65	M	R	Hemorrhagic	Left thalamus	10
4	67	M	R	Ischemic	Left MCA	26
5	71	M	R	Ischemic	Right MCA	15
6	58	F	R	Ischemic	Right MCA	35
7	56	F	R	Ischemic	Left MCA	27
8	52	F	R	Hemorrhagic	Left thalamus	17
9	60	M	R	Hemorrhagic	Right putamen	79
10	66	M	R	Hemorrhagic	Right thalamus	7
*Mean±SD*	*61.1±7.1*					*25.2±20.1*

R: right, ACA: anterior cerebral artery, and MCA: middle cerebral artery.

**Table 2:** Paretic upper limb activity and upper limb function.

Case	MAL	Accelerometer	FMA-UE			ARAT
	AOU	Laterality index	Total	Proximal	Distal	Total
	(0–5)	(-1 to +1)	(0–66)	(0–42)	(0–24)	(0–57)

*Baseline*						
1	2.8	-0.16	66	42	24	57
2	1.0	-0.60	50	37	13	37
3	0.5	-0.22	55	34	21	34
4	1.1	-0.38	45	35	10	23
5	1.6	-0.42	61	37	24	53
6	2.2	-0.40	52	37	15	26
7	3.1	-0.07	61	37	24	56
8	0	-0.73	43	20	23	22
9	0	-0.37	15	15	0	3
10	0.3	-0.32	51	33	18	26
*Mean±SD*	*1.3±1.1*	*-0.37±0.2*	*49.9±13.5*	*32.8±8.0*	*17.2±7.5*	*33.7±16.5*

*Post*						
1	3.7	-0.17	66	42	24	57
2	1.3	-0.42	51	38	13	39
3	1.0	-0.22	57	36	21	42
4	1.5	-0.34	45	35	10	23
5	3.0	-0.23	62	38	24	54
6	2.5	-0.40	53	38	15	27
7	3.4	-0.02	61	37	24	57
8	0.7	-0.54	43	20	23	26
9	2.6	-0.35	15	15	0	3
10	1.1	-0.27	51	33	18	29
*Mean±SD*	2.1^*∗*^*±1.0*	−0.30^*∗*^*±0.1*	*50.4±13.7*	*33.2±8.2*	*17.2±7.5*	35.7^*∗*^*±16.5*

*Follow-up*						
1	4.3	-0.18	66	42	24	57
2	1.3	-0.31	51	38	13	41
3	0.9	-0.33	56	35	21	42
4	1.7	-0.34	45	35	10	23
5	3.9	-0.17	62	38	24	53
6	2.5	-0.32	52	37	15	28
7	3.9	-0.06	61	37	24	57
8	0.6	-0.70	43	20	23	27
9	1.2	-0.37	15	15	0	3
10	1.4	-0.32	48	30	18	33
*Mean±SD*	2.2^*∗*^*±1.3*	*-0.31±0.2*	*49.9±13.7*	*32.7±8.3*	*17.2±7.5*	36.4^*∗*^*±16.3*

MAL: Motor Activity Log, AOU: amount of use, accelerometer: arm triaxial accelerometer, FMA-UE: Fugl–Meyer assessment-upper extremity, and ARAT: action research arm test; ^*∗*^*p* < 0.05 (Wilcoxon signed-rank test).

## Data Availability

The data used to support the findings of this study are available in the Supplementary Materials.

## Supplementary Materials

Supplementary Materials S1 Table: Motor Activity Log (MAL; mean range 0–5; amount of use (AOU)). S2 Table: method of calculation for accelerometry (laterality index (LI)). S3 Table: Fugl–Meyer assessment- (FMA-) upper extremity (FMA-UE; score range, 0–66). S4 Table: action research arm test (ARAT; score range, 0–57).
