# Supplementary material for: Effects of Home-Based Robotic Therapy Involving the Single-Joint Hybrid Assistive Limb Robotic Suit in the Chronic Phase of Stroke: A Pilot Study
Source: Biomed Res Int. 2019 Mar 18;2019:5462694. doi: 10.1155/2019/5462694 (PMC6442446; doi:10.1155/2019/5462694)
Supplement: Supplementary Materials — S1 Table: Motor Activity Log (MAL; mean range 0–5; amount of use (AOU)). S2 Table: method of calculation for accelerometry (laterality index (LI)). S3 Table: Fugl–Meyer assessment- (FMA-) upper extremity (FMA-UE; score range, 0–66). S4 Table: action research arm test (ARAT; score range, 0–57). [file 5462694.f1.docx]

Data are freely available,

we ask that authors note this and state the location of their data:

- Within the paper, supporting information files, in a public repository

**Supporting information**

**S1 Table. Motor Activity Log (MAL; mean range 0–5; amount of use [AOU]).**

**S2 Table. Method of calculation for accelerometry (laterality index [LI]).**

**S3 Table. Fugl–Meyer assessment (FMA)-upper extremity (FMA-UE; score range, 0–66).**

**S4 Table. action research arm test (ARAT; score range, 0–57).**

**S1 Table. Motor Activity Log (MAL; mean range 0–5; amount of use [AOU]).**

**S2 Table. Method of calculation for accelerometry (laterality index [LI]).**

**S3 Table. Fugl–Meyer assessment (FMA)-upper extremity (FMA-UE; score range, 0–66)**

**S4 Table. action research arm test (ARAT; score range, 0–57)**
